# Phospholipids with two polyunsaturated fatty acyl tails: an important driver of ferroptosis

**DOI:** 10.1002/mco2.606

**Published:** 2024-06-25

**Authors:** Chu Lu, Xiaoxue Zhou, Long Zhang

**Affiliations:** ^1^ Life Sciences Institute, The MOE Key Laboratory of Biosystems Homeostasis & Protection and Zhejiang Provincial Key Laboratory for Cancer Molecular Cell Biology The Second Affiliated Hospital of the Zhejiang University School of Medicine Zhejiang University Hangzhou China; ^2^ The MOE Basic Research and Innovation Center for the Targeted Therapeutics of Solid Tumors The First Affiliated Hospital, Jiangxi Medical College Nanchang University Nanchang China; ^3^ Cancer Center Zhejiang University Hangzhou China

**Keywords:** ferroptosis, phospholipid

## Abstract

We highlight the latest work of Qiu et al. on the core mechanism of ferroptosis induced by rare phospholipids with two polyunsaturated fatty acyl tails (PL‐PUFA_2_s), which has been published in *Cell*. It has long been acknowledged that PLs containing one PUFA tail (PL‐PUFA_1_s) serve as substrates for phospholipid peroxidation during the process of ferroptosis, owing to their susceptibility to oxidation and prevalence in vivo. However, the authors note that PL‐PUFA_2_s, rather than PL‐PUFA_1_s, represent critical lipid classes involved in the pro‐ferroptosis process. Exogenous phosphatidylcholine‐PUFA_2_s accumulate in mitochondria and combine with Complex I within the electron transport chain, thereby potentially resulting in an elevation of mitochondrial reactive oxygen species levels. Then, these mitochondrial peroxides prompt the substantial accumulation of peroxides within the endoplasmic reticulum, ultimately culminating in ferroptosis. These findings shed light on the potential molecular mechanisms underlying the induction of ferroptosis by dietary PL‐PUFA_2_s and offer novel insights for both the evaluation of cellular iron death sensitivity and the treatment of cancer. This article will provide a more comprehensive elucidation of the paper and facilitate an enhanced understanding of the underlying mechanisms for readers.

## Graphical abstract

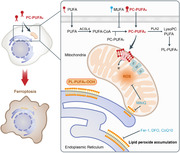

1

In a recently published study in *Cell*,^1^ a group led by Brent R. Stockwell performed a detailed investigation of the relationship between Phospholipids harboring PUFA tails at both the sn1 and sn2 positions (PL‐PUFA_2_s) and ferroptosis. They found that dietary PL‐PUFA_2_s could induce the initiation and propagation of lipid peroxidation that contributed to ferroptosis (Figure 1).

**FIGURE 1:**
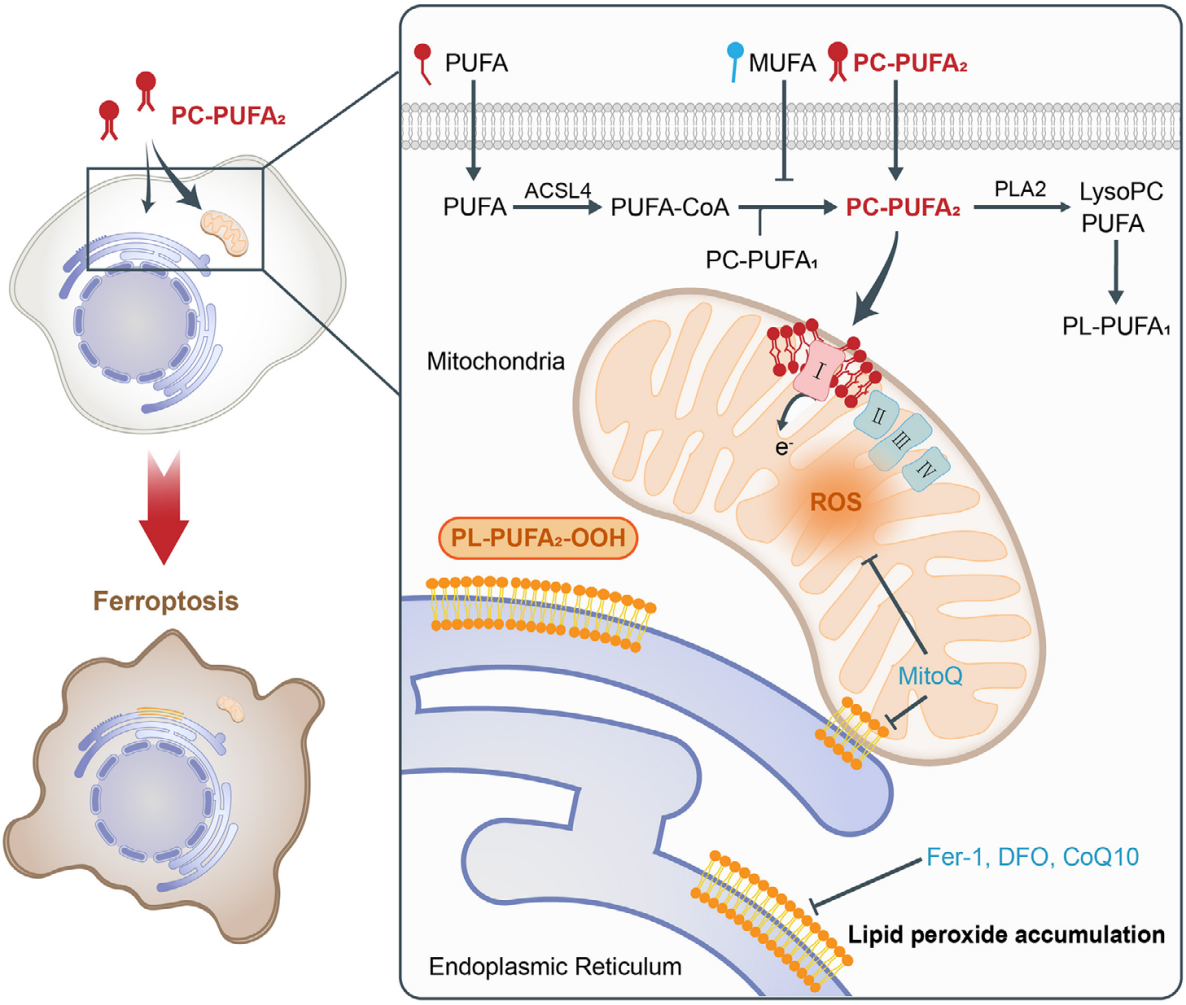
The mechanism by which dietary phosphatidylcholine with two polyunsaturated fatty acyl tails (PC‐PUFA_2_s) promotes ferroptosis. When exogenous PC‐PUFA_2_s enter the cell, only a portion of these lipids are degraded by phospholipase A2 (PLA2), and the rest accumulate significantly in mitochondria and endoplasmic reticulum (ER), especially mitochondria. PC‐PUFA_2_s in mitochondria bind to electron transport chain (ETC) complex I, concomitant with an elevation in mitochondrial reactive oxygen species (mtROS) levels and the accumulation of lipid peroxides. Lipid hydroperoxides (PL‐PUFA‐OOHs) originating from mitochondria can communicate with the ER membrane through the mitochondria‐ER contact site, which may be responsible for lipid peroxide accumulation in the ER, ultimately leading to ferroptosis. Additionally, exogenous PUFAs are the raw material for the synthesis of PC‐PUFA_2_s in vivo, thereby inducing ferroptosis, whereas exogenous monounsaturated fatty acids (MUFAs) inhibit the PC‐PUFA_2_s synthesis pathway, and exert the opposite effect.

Ferroptosis is an iron‐dependent non‐apoptotic form of cell death and excess iron has been reported to promote phospholipid peroxidation and mitochondrial dysfunction.^2^ PLs containing one PUFA tail (PL‐PUFA_1_s) are characterized by high abundance within mammalian cells and the presence of di‐acyl allyl groups within the PUFA tails renders them susceptible to oxidation. Therefore, PL‐PUFA_1_s are believed to actively participate in lipid peroxidation, leading to ferroptosis. However, a recent discovery revealed that protecting PL‐PUFA_2_s, rather than PL‐PUFA_1_s, from oxidative degradation could confer ferroptosis resistance to cells.^3^ This interesting finding caught the attention of Qiu et al. and they subsequently conducted substantial experiments to explore the function of PL‐PUFA_2_s in cell death.

Initially, Qiu et al. proved there existed a strong correlation between PL‐PUFA_2_s and ferroptosis. They observed that although many types of PLs were capable of causing cell death, phosphatidylcholines (PCs), especially PC‐PUFA₂s, exhibited the most specific ability to induce ferroptosis among them. Previous studies showed that accumulation of oxidized PE‐PUFA precipitated ferroptosis. However, in this study, the supplementation of PE‐PUFA was less effective than PC‐PUFA in inducing ferroptosis. The authors suggested that this may stem from the difficult uptake of exogenous PE‐PUFA, coupled with its heightened cytotoxicity. The elevation of ferroptosis biomarkers, accrual of lipid peroxides, and observed synergistic effects with established ferroptosis inducers further corroborated the ferroptosis‐inducing potential of PL‐PUFA_2_s. Moreover, liquid chromatography (LC)‐mass spectrometry (MS)‐based targeted lipidomics substantiated the correlation between the abundance of PC‐PUFA_2_s and cellular susceptibility to ferroptosis. In aging and degenerative diseases, iron accumulation contributes to vulnerability to lipid oxidation and consequential ferroptosis. The authors found that PL‐PUFA_2_ was the main substrate for lipid oxidation in the hippocampus of senescent mice and the brain tissue of patients with Huntington's disease, indicating that the apparent depletion of PL‐PUFA_2_s might serve as a marker of ferroptosis.

Then, Qiu et al. explored the role of exogenous PL‐PUFA_2_s in cellular systems. By extracting and analyzing lipids from the cells treated with PL‐PUFA_2_s, they found that in contrast to PL‐PUFA_1_s, exogenous PL‐PUFA_2_s exhibited enhanced stability within the cellular environment, potentially explaining the higher ferroptosis inducibility of exogenous PL‐PUFA_2_s. Furthermore, by employing isotope labeling of the fatty acyl tails of exogenous PC‐PUFA_2_s, researchers observed their successful incorporation into cellular membranes, instigating lipid remodeling processes.

Previous research revealed that free PUFAs promoted ferroptosis while free monounsaturated fatty acids (MUFAs) inhibited ferroptosis, but the mechanism was unclear. In this study, Qiu et al. provided one proper explanation by conducting experiments. The co‐treatment with free PUFAs had a larger synergistic effect with PC‐PUFA_1_s than PC‐PUFA_2_s. Based on this phenomenon, they postulated that the interaction between PC‐PUFA_1_s and free PUFAs may result in the formation of PC‐PUFA_2_s, thereby exacerbating cell death. This hypothesis was partially substantiated by a marked increase in the content of PC‐PUFA_2_s in cells treated with free PUFAs. Another finding that free MUFAs decreased the levels of PC‐PUFA_2_s demonstrated that MUFAs hampered the synthesis of PC‐PUFA_2_s. Additionally, the authors proved that PC‐PUFA_2_s were proximal mediators of ferroptosis.

The above results prompted Qiu et al. to explore the mechanism underlying PC‐PUFA_2_s‐induced ferroptosis. Employing the affinity pull‐down assay with MS, they observed that PL‐PUFA_2_s exhibited a specific binding affinity towards mitochondrial electron transport chain (ETC) complex I, in alignment with outcomes derived from gene set enrichment analysis (GSEA). Numerous studies have indicated mitochondrial phospholipid composition, particularly cardiolipin (CL) and Ether phospholipids, had a significant impact on the function of ETC complex I. However, investigations into the interaction of PLs with specificity in the tail with complex I are still scant. It is conceivable that PL‐PUFA_2_s possess the capability to bind to specific sites within the structural domains of complex I, akin to CL, and cause protein conformational changes, ultimately resulting in increased reactive oxygen species (ROS) production. Subsequently, the co‐staining experiment and LC‐MS analysis revealed a heightened accumulation of exogenous lipids within both the mitochondria and the endoplasmic reticulum (ER). This observation may result from the prevalence of endogenous PL‐PUFA_2_ trafficking mechanisms within mitochondria, a phenomenon hitherto found in yeast but not yet identified in mammalian cells. These investigations collectively underscored the pivotal role of mitochondria as a significant site for the accumulation of PC‐PUFA_2_s, further emphasizing their involvement in the induction of ferroptosis.

Therefore, the authors assessed the potential impact of PC‐PUFA_2_s accumulation on mitochondrial physiology. The presence of PC‐PUFA_2_s resulted in elevated levels of mitochondrial ROS (mtROS), accompanied by a decline in mitochondrial membrane potential and dampening of mitochondrial respiration. This finding underscored the ability of PC‐PUFA_2_s to induce mitochondrial oxidative stress. However, the addition of other ETC complex inhibitors caused mitochondrial oxidative stress but failed to induce ferroptosis, and a similar phenomenon appeared in one prior research.^4^ They collectively suggest the significant role of phospholipids and in this study PL‐PUFA_2_ might induce mtROS production, concurrently serving as the substrate for mtROS oxidation. Consequently, Qiu et al. evaluated the role of mtROS in PC‐PUFA_2_‐induced ferroptosis. The utilization of mitochondria‐targeted ROS scavengers effectively mitigated lipid peroxidation and ferroptosis, demonstrating the significance of mtROS. Moreover, the potency of PC‐PUFA_2_s in inducing ferroptosis was diminished in cells with reduced mitochondrial content. However, in these cells, spare accumulation of PC‐PUFA_2_s within organelles such as the ER might serve as the primary initiator of the lethal effect.

Co‐staining of cells with fluorescent probes targeting lipid peroxides along with organelle markers revealed a notable increase in lipid peroxide levels after treatment with PC‐PUFA_2_s, particularly in ER. This demonstrated that ER was the principal site of lipid peroxidation. However, further experiments did not find any evidence of ER stress following treatment with PC‐PUFA_2_s.

Qiu et al. pioneered the elucidation of the significant ability of dietary PL‐PUFA_2_s to induce ferroptosis. Their seminal work revealed the mechanism through which dietary PL‐PUFA_2_s, especially PC‐PUFA_2_s, promote ferroptosis (Figure 1). Numerous PC‐PUFA_2_s are absorbed by cells and subsequently undergo lipid remodeling. Intriguingly, these PC‐PUFA_2_s predominantly accumulate in mitochondria and interact with ETC complex I, thereby elevating mtROS levels. Consequent mitochondrial peroxide production precipitates the accumulation of lipid peroxidation within the ER through mitochondria‐ER contact sites, ultimately inducing ferroptosis. In addition, the authors found that PL‐PUFA_1_ could bind to peroxisome‐related proteins. Considering PUFA has been proven to interact and activate the peroxisome proliferator‐activated receptors (PPARs)^,^ ^5^ we speculate that a similar mechanism may exist for PL‐PUFA_1_, which needs to be further explored.

Although Qiu et al. have outlined a foundational framework for understanding the ontogeny of ferroptosis induced by dietary lipids, particularly PL‐PUFA_2_s, there exist several challenges that must be addressed by future studies. These include the specific pathways through which dietary MUFAs modulate the synthesis of PL‐PUFA_2_s in vivo, the exact binding site of PC‐PUFA_2_s within ETC complex I, and so on.

In summary, this latest research has significantly advanced our understanding of the intricate interplay between ferroptosis and lipid metabolism. Additionally, PL‐PUFA_2_ may be established as the discerning marker for evaluating cellular vulnerability to ferroptosis.

## AUTHOR CONTRIBUTIONS

C.L. wrote the manuscript and prepared the figure. X.Z. provided valuable discussion. L.Z. approved the final version of the manuscript. All authors have read and approved the final manuscript.

## CONFLICT OF INTEREST STATEMENT

The authors declare no conflict of interest.

## FUNDING INFORMATION

Not applicable.

## ETHICS STATEMENT

Not applicable.

## Data Availability

Not applicable.
